# Cybervictimization and cyberbullying among college students: The chain mediating effects of stress and rumination

**DOI:** 10.3389/fpsyg.2023.1067165

**Published:** 2023-02-10

**Authors:** Qing Luo, Na Wu, Lu Huang

**Affiliations:** ^1^Department of Psychology, School of Public Policy and Administration, Nanchang University, Nanchang, China; ^2^Department of Psychology, College of Education and Science, Hubei Normal University, Huangshi, China; ^3^School of Marxism, Wuhan Business University, Wuhan, China

**Keywords:** cybervictimization, cyberbullying, stress, rumination, college students

## Abstract

The popularity of the Internet has led to an increase in cybervictimization and cyberbullying. Many studies have focused on the factors influencing cybervictimization or cyberbullying, but few have researched the mechanism that mediates these phenomena. Therefore, in this study, we use a chain mediation model to explore the mechanisms of cybervictimization and cyberbullying. This research is based on the general aggression model and examines whether stress and rumination play a mediating role in the relationship between cybervictimization and cyberbullying among Chinese college students. This study included 1,299 Chinese college students (597 men and 702 women, *M* = 21.24 years, *SD* = 3.16) who completed questionnaires on cybervictimization, stress, rumination, and cyberbullying. Harman’s one-factor test was used to analyze common method bias; mean and standard deviations were used to analyze the descriptive statistics, Pearson’s moment correlation was used to determine the relationship between variables, and Model 6 of the SPSS macro examined the mediating effect of stress and rumination. The results indicate that rumination mediated the relationship between cybervictimization and cyberbullying. In addition, stress and rumination acted as a chain mediator in this association. These results have the potential to reduce the likelihood of college students engaging in cyberbullying as a result of cybervictimization, minimize the rate of cyberbullying among youths, and lead to the development of interventions for cybervictimization and cyberbullying.

## 1. Introduction

The Internet plays an important role in people’s lives; however, there are risks associated with using the Internet, such as cybervictimization and cyberbullying (Ferrara et al., 2018). It is obvious that the proliferation of the internet has resulted to an increased cases of cybervictimization and cyberbullying, which have become prevalent in society (Ding et al., 2020). Studies from the United States, the United Kingdom, Switzerland, and Turkey have demonstrated a strong correlation between increased Internet use and increased cybervictimization and cyberbullying (Hinduja and Patchin, 2008; Smith et al., 2008; Sticca et al., 2013). Cybervictimization and cyberbullying have become a major concern for college students (Khine et al., 2020; Martínez-Monteagudo et al., 2020; Qudah et al., 2020), and several recent studies have investigated cyberbullying behavior among college students (Alrajeh et al., 2021; Lam et al., 2022).

Cyber-victims are those affected by cyberbullying (Betts, 2015), and cybervictimization usually occurs through electronic media (Tokunaga, 2010). Cybervictimization is widespread, with a large and serious scope of abuse (Dempsey et al., 2009). Ansary (2020) analyzed numerous studies and found that the average annual cybervictimization rate was 14–21%. Globally, 10 to 72% of youths have reported being victims of cyberbullying (Tokunaga, 2010; Mishna et al., 2011). Most adolescents who are bullied online experience mental health problems, including stress and maladaptive regulation strategies (Tokunaga, 2010; Albdour et al., 2017; Palermiti et al., 2017; Musharraf et al., 2019), some have even committed suicide (Quintana-Orts et al., 2022). Cyber-victims experience bullying multiple times and are more likely to be involved in cyberbullying (Zhu et al., 2019; Dong, 2020; Wang et al., 2020). Faucher et al. (2014) found that of the 24.1% of Canadian university students who experienced cyberbullying, 5.1% engaged in cyberbullying.

Cybervictimization is more likely to lead to cyberbullying, thus creating a vicious cycle (Sun et al., 2020). Many studies have found a strong correlation between cybervictimization and cyberbullying (Leung et al., 2018; Lozano-Blasco et al., 2020). A meta-analysis based on cyberbullying found a significantly positive correlation between cybervictimization and cyberbullying (Kowalski et al., 2014). Moreover, cybervictimization has been identified as a strong predictor of cyberbullying (Kwan and Skoric, 2013; Kowalski et al., 2014). Cyber-victims are at high risk of becoming cyberbullies (Walrave and Heirman, 2011; Hemphill et al., 2012). Some cyber-victims might respond to cyberbullying with cyberbullying behavior (Yilmaz, 2011). Cybervictimization is the strongest predictor of cyberbullying (Akbulut and Eristi, 2011). Dehue et al. (2008) found that 5.7% of cyberbullied adolescents chose a retaliatory coping strategy, such as cyberbullying. Cyber-victims often commit cyberbullying in the same online environment where they experience bullying (Gradinger et al., 2010). A longitudinal study of teenagers from four Midwestern U.S. middle schools found that teens who had been bullied online demonstrated aggressive behavior (e.g., cyber relational or verbal aggression) 6 months later (Wright and Yan, 2013). Chu et al. (2018) found that previous cybervictimization experiences positively predicted subsequent cyberbullying behavior, and Espelage et al. (2012) argued that cyber-victims would perpetrate cyberbullying.

Cyberbullying is an intentional act repeatedly committed against an individual or a group by an individual or group using electronic information communication tools (Smith et al., 2008; Menesini et al., 2012; Jadambaa et al., 2019). The incidence of cyberbullying is increasing with the continuous development of Internet technology (Yıldız Durak, 2019). Cyberbullying is a serious global social problem that affects individuals who access the Internet or mobile networks regardless of age, education, and socioeconomic problems (Akbulut and Eristi, 2011; Garaigordobil and Martínez-Valderrey, 2015; Festl, 2016). Cyberbullying is common among college students with an incidence rate of 10–50% (Kowalski et al., 2012; Kokkinos et al., 2014). Compared to individuals at other ages, college students are more likely to engage in cyberbullying because they can use the Internet for long periods of time and unsupervised, as well as frequently showcase their lives on social media, seek experiences, and form social cliques (Jones and Scott, 2012). A study of Turkish university students demonstrated that 59.8% of undergraduates engaged in cyberbullying (Turan et al., 2011). A meta-analysis study on bullying prevalence across contexts revealed that the average incidence of cyberbullying was about 15%; however, the study included traditional bullying, and the study only focused on peer cyberbullying (Modecki et al., 2014). According to UNICEF study, 19.7% college students reported participating in cyberbullying at least once in their lifetime, and 54.4% reported experiencing cyberbullying at least once in their lifetime (Ozden and Icellioglu, 2014).

The general aggression model is considered a valuable theoretical framework for explaining cyberbullying among college students (Wong et al., 2018). The model (Bushman and Anderson, 2002) provides a comprehensive theoretical framework that includes both individual-specific and situation-specific factors that can be effectively used to explain cybervictimization and cyberbullying. The general aggression model suggests that the occurrence of aggression includes three processes: an individual and situational input variable process, a path process, and an output variable process. According to the generalized aggression model, the cyberbullying experience is an individual and situational input variable process that changes the individual’s state and drives aggressive behavior. Cybervictimization acts as a trigger for individuals to instigate cyberbullying behaviors (Wong et al., 2018). Lang (1968) proposed that aversive events awaken an individual’s hostile attitude and eventually provoke the impulse to engage in aggressive behavior. The impulses triggered by aversive events are also the strongest situational triggers (Finkel and Eckhardt, 2013).

Most extant studies have focused on the relationship between traditional bullying and cyberbullying (Tomazin and Smith, 2007; Bhat, 2008; Doneman, 2008) and the influencing factors of cybervictimization and cyberbullying such as gender, emotional problems, depression, anxiety, and other physical and psychosomatic problems (Desmet et al., 2014; Gimenez Gualdo et al., 2015; Yildirim et al., 2019). However, the mechanisms underlying the mediating or moderating factors between cybervictimization and cyberbullying remain unclear. The general aggression model explains the occurrence of cyberbullying from both person-specific and situation-specific factors. Therefore, this study applies this theory to analyze the mechanism of the association between cybervictimization and cyberbullying.

### 1.1. The role of stress as a mediator between cybervictimization and cyberbullying

According to the general aggression model, stress is a pathway process that affects an individual’s current cognitive and affective states, which in turn stimulate the individual’s physiological state. Stress is a cacoethic state that has harmful effects on the mind and body (Weiten et al., 2014). Stress is also an important correlate of aggressive tendencies in college students (Velezmoro et al., 2010). Within the social information processing framework, research has primarily investigated the mechanisms that link this stressor to simultaneous and future aggressive behaviors (Dodge, 1993). The experience of being cyberbullied can lead to stress (Monks et al., 2012). Cyber-victims have reported symptoms of stress (Williams et al., 2017). Snyman and Loh (2015) demonstrated that cyber-victims might experience stress, while Martínez-Monteagudo et al. (2020) showed that cyber-victims could exhibit high levels of stress. González-Cabrera et al. (2017) measured stress perceptions based on cortisol and found that cybervictimization events induce stress. Many studies have found a positive correlation between cybervictimization and stress (Martins et al., 2016), including an association with high levels of social stress (Fredstrom et al., 2011), and a meta-analysis study indicated that stress is very highly correlated with cybervictimization (Kowalski et al., 2014). Peer victimization is a significant stressor for adolescents, and victimized adolescents are more likely to develop aggressive behaviors than adolescents who are not victimized (Prinstein et al., 2005). Patchin and Hinduja (2011) argued that as a response to stressful life events, some young people may engage in bullying behaviors (both traditional and online). In addition, Lianos and Mcgrath (2018) revealed that cybervictimization, as a negative stimulus, was an important stressor that led to cyberbullying and that adolescents were more likely to exhibit cyberbullying behaviors after experiencing stressful events. Garaigordobil and Machimbarrena (2019) confirmed positive correlations among cybervictimization, cyberbullying, and stress. The experience of cybervictimization will affect cyber victims’ responses to stress, and they might become involved in cyberbullying (Kowalski et al., 2014).

### 1.2. The mediating role of rumination between cybervictimization and cyberbullying

According to the general aggression model, rumination is another pathway process, and rumination affects an individual’s current cognitive and affective states, which in turn stimulate the individual’s physiological state. Rumination is considered an emotion regulation strategy in which individuals repetitively focus on the reasons, consequences, and meanings of negative emotions (Nolen-Hoeksema, 1991). A victimization environment may influence an individual’s sense of self so that they attribute the bullying to their own personality or behavior, thus engaging in self-blame, a form of rumination (Graham and Juvonen, 2001). Victimization is related to self-blaming attributions (Taylor et al., 2013); after being bullied, individuals will attribute the cause to themselves, which leads to rumination. Cybervictimization has been found to be positively correlated with rumination (Feinstein et al., 2014; Rey et al., 2020). Zhong et al. (2015) demonstrated that cybervictimization was perceived as a negative life event, leading many junior high school students to wonder why they were always bullied; moreover, they showed that it positively predicted rumination.

Negative thinking can cause negative behaviors, and high levels of rumination can induce aggressive behavior (Zhu, 2014; Zhong et al., 2015). According to Feinstein et al. (2014), the tendency to ruminate was elevated after exposure to online violence. Rumination can significantly and accurately predict a variety of aggressive behaviors (Peters et al., 2015), and cyberbullying is a sub-category of aggressive behavior (Smith et al., 2008).

### 1.3. The chain mediating role of stress and rumination between cybervictimization and cyberbullying

Victimization experiences may promote ruminative responses to social stress (Miernicki, 2015). People facing chronic highly intense sources of stress and factors beyond their control may adopt ruminative behaviors (Nolen-Hoeksema and Girgus, 1994), which can be amplified in the presence of stress (Morrison and O’Connor, 2005). Numerous studies have demonstrated that rumination is influenced by stress (Nolen-Hoeksema et al., 2008). More stressful events cause higher levels of negative emotions; therefore, individuals repeatedly think about ways to reduce stress to reduce the level of negative emotions, leading to ruminant thinking (Guo et al., 2011).

### 1.4. The present study

**Figure 1:**
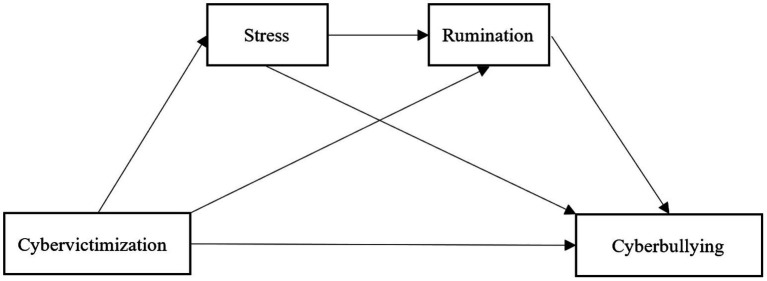
Theoretical model.

To effectively reduce cyberbullying, it is crucial to explain the factors influencing various aspects of cyberbullying (Musharraf et al., 2019; Qudah et al., 2020; Türk et al., 2021). Therefore, this study constructs a sequential mediation model that is based on the general aggression model. In the study model, cybervictimization is an individual and situational input variable process, stress and rumination are path processes, and cyberbullying is an output variable process. As mentioned above, cybervictimization is one of the stressors (Lianos and Mcgrath, 2018), and stress and rumination lead to risk factors for cyberbullying (Kowalski et al., 2014; Peters et al., 2015). In addition, cyber-victims report perceived stress; as there is a positive correlation between perceived stress and cybervictimization, stress-motivated individuals are more likely to engage in cyberbullying behaviors. Therefore, based on the general aggression model, a chain mediation model is used, and the following three hypotheses are proposed (Figure 1):

*Hypothesis 1*: Stress mediates the relationship between cybervictimization and cyberbullying.

*Hypothesis 2*: Rumination mediates the relationship between cybervictimization and cyberbullying.

*Hypothesis 3*: Stress and rumination play a sequential mediating role in the relationship between cybervictimization and cyberbullying.

## 2. Materials and methods

### 2.1. Participants

A convenience sampling technique was used to select 1,335 undergraduates from two universities in East China through. Of the total sample, 597 were males and 702 were females. Freshmen, sophomores, juniors, and seniors were 297, 315, 246 and 441, respectively. The participants’ ages ranged from 18 to 24 years (*M* = 21.24, *SD* = 3.16). A total of 36 participants were excluded from the questionnaire because they could not respond within the short response time. Finally, 1,299 valid questionnaires were obtained (return rate of 97.3%).

### 2.2. Measures

#### 2.2.1. Cybervictimization

The Chinese version of the cybervictimization scale was used to measure cybervictimization (Chu and Fan, 2017). The scale comprises 14 items measuring how often the participants experienced cyberbullying through various channels, such as QQ, Weibo, and WeChat, over 6 months. An example of the questionnaire items is, “Someone abused me online (e.g., QQ, WeChat, Weibo, chat rooms, RenRen, etc.).” The items were rated using a four-point Likert-type scale (1 = “never experienced” to 4 = “experienced more than 3 times”), where a higher score indicated more frequent cybervictimization experiences. The Cronbach’s alpha coefficient in the present study was 0.94.

#### 2.2.2. Stress

The stress sensitivity scale was used to assess the level of stress (Li and Mei, 2002). The scale comprises 15 items regarding stress experienced after cyberbullying events. An example of the questionnaire items is “Someone sent me a threatening or hurtful text message.” The items were rated on a four-point Likert-type scale (1 = “no stress” to 4 = “severe stress”), where a higher score indicated a higher level of stress. The Cronbach’s alpha in the present study was 0.96.

#### 2.2.3. Rumination

The ruminant thinking scale was used to measure the degree of ruminative thinking induced by negative life events in general (Han and Yang, 2009). An example of the questionnaire items is “I often wonder what I have done to cause this.” The scale comprises 22 items rated on a four-point Likert-type scale (1 = “never” to 4 = “always”), where higher scores indicate more severe ruminative thinking. The Cronbach’s alpha coefficient in the present study was 0.97.

#### 2.2.4. Cyberbullying

The Chinese version of the cyberbullying questionnaire (Chu and Fan, 2017) was used to measure how often the participants engaged in cyberbullying behavior, such as ostracizing someone online by limiting and deleting comments, over 6 months. An example of the questionnaire items is “Abuse someone online (e.g., QQ, WeChat, Weibo, chat rooms, RenRen, etc.).” The scale comprises 14 items rated on a four-point Likert-type scale (1 = “never implemented” to 4 = “implemented more than 3 times”), where a higher score indicated more frequent cyberbullying experiences. The Cronbach’s alpha coefficient in the present study was 0.97.

### 2.3. Procedure and data analysis

Before the questionnaires were distributed, three psychology and cyberpsychology experts were invited to evaluate the questionnaire to ensure that the content would not affect the participants. Participants were recruited *via* QQ, WeChat, and school forums, and completed the questionnaire *via* Wenjuanxing’ platform. The participants completed the questionnaire online, and prior to participation, privacy and confidentiality were assured, informed consent was obtained, and the instructions were clearly articulated. Before completing the questionnaire, the participants were told that there were no right or wrong answers.

The data were collected between May and June 2021. Class instructors were contacted in advance to determine a time to complete the questionnaire online. The average time to complete the questionnaire was approximately 15 min.

The collected data were subjected to a stepwise analysis with the aid of statistical software, including IBM SPSS 25.0 and PROCESS. First, we used Harman’s one-factor test for common method bias for the original data; second, the mean and standard deviations of all variables were analyzed; third, the Pearson’s correlation coefficients between all variables were determined; then, we examined the mediating effect of stress and rumination using Model 6 of the SPSS macro developed by Hayes (2017). We estimated the 95% confidence intervals of the mediating effect with 5,000 resamples.

## 3. Results

### 3.1. Common method bias

To effectively control for common method bias, the participants were informed of the anonymity and rigor of the questionnaires; to promote truthful responses, they were also assured that information would not be disclosed. An exploratory factor analysis was conducted using Harman’s one-way method to examine the items for common method bias. The results indicated that there was no common method bias in this study. There were 17 factors with eigenvalues greater than 1, with 28.52% of the variance explained by the first factor, which was less than the critical criterion of 40% (Zhou and Long, 2004).

### 3.2. Mean, standard deviation, and correlation analysis of each variable

The results revealed significant positive correlations among cybervictimization, stress, rumination, and cyberbullying (Table 1).

**Table 1:** Means, standard deviations, and correlations between cybervictimization, stress, rumination, and cyberbullying.

Variable	*M*	*SD*	1	2	3	4
Cybervictimization	1.71	0.71	1			
Stress	1.86	0.81	0.70^**^	1		
Rumination	1.80	0.76	0.76^**^	0.84^**^	1	
Cyberbullying	1.53	0.77	0.92^**^	0.71^**^	0.79^**^	1

*N* = 1,299. ^**^p < 0.01.

### 3.3. Chain mediation analysis

Controlling for gender variables, the chain mediation model in the PROCESS plugin was used to examine the chain mediation between stress and rumination (Table 2).

**Table 2:** Regression analysis of the relationship between the variables in the chain mediation model.

Regression equation (*N* = 1,299)		Overall fit index			Significance of regression coefficients	
Result variables	Predictive variables	*R*	*R^2^*	*F*	*β*	*T*
Stress	Cybervictimization	0.73	0.53	97.41^**^	0.82	21.27^***^
Rumination	Stress	0.88	0.78	256.38^***^	0.52	16.76^***^
	Cybervictimization				0.42	11.79^***^
Cyberbullying	Stress	0.93	0.86	377.03^***^	0.01	0.18
	Rumination				0.23	5.80^***^
	Cybervictimization				0.79	24.02^***^

^**^p < 0.01; ^***^p < 0.001.

Table 2 presents the overall path coefficients of the mediation analysis. The results indicated that cybervictimization positively predicted stress (*β* = 0.82, *p* < 0.001), and there was a significant predictive effect of stress for rumination (*β* = 0.52, *p* < 0.001). Cybervictimization significantly predicted rumination (*β* = 0.42, *p* < 0.001), and cybervictimization positively predicted cyberbullying (*β* = 0.79, *p* < 0.001). Rumination significantly predicted cyberbullying (*β* = 0.23, *p* < 0.001); however, stress was not a significant predictor of cyberbullying (*β* = 0.01, *p* > 0.05).

**Figure 2:**
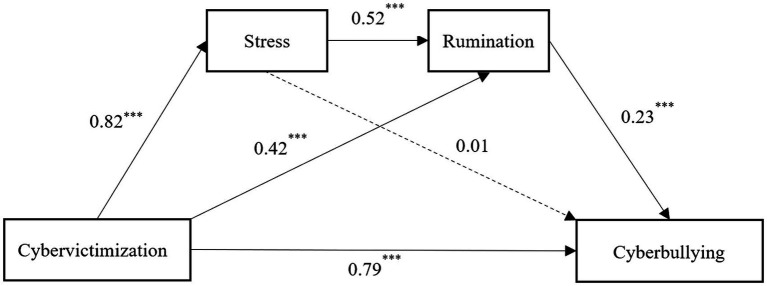
Model of the chain-mediating effects of stress and rumination on the relationship between cybervictimization and cyberbullying.

The bias-corrected nonparametric bootstrap method was used to test for the mediation effect in this study. The test for mediation effects was performed with 5,000 replicate samples, and 95% confidence intervals were calculated (Table 3). The results revealed two pathways with significant indirect effects: (1) cybervictimization → stress → rumination → cyberbullying, with an indirect effect value of 0.09, indicating that stress and rumination were significant in cybervictimization and cyberbullying, and (2) cybervictimization → rumination → cyberbullying, with an indirect effect value of 0.09, indicating that rumination partially mediated the relationship between cybervictimization and cyberbullying (Figure 2).

**Table 3:** Standardized indirect effects from stress and rumination.

	*β*	Boot SE	Boot confidence interval lower limit	Boot confidence interval upper limit
Total indirect effect	0.19	0.03	0.14	0.27
*Via* stress	0.005	0.03	−0.06	0.07
*Via* stress and rumination	0.09	0.02	0.06	0.15
*Via* rumination	0.09	0.03	0.05	0.15

## 4. Discussion

This study built on the existing literature to clarify the roles of stress and rumination in cybervictimization and cyberbullying. The results of this study support the hypotheses that rumination plays a mediating role in the relationship between cybervictimization and cyberbullying and that stress and rumination act as chain mediators.

The first hypothesis proposed that stress plays a mediating role in the relationship between cybervictimization and cyberbullying. However, this hypothesis was not supported by this study’s results, nor were the results consistent with Lianos and Mcgrath (2018). There could be multiple reasons for this result. According to Agnew’s (1992) general stress theory, stress from cyberbullying causes negative emotions and can lead to bullying others or increase the level of delinquent adaptations (Mazerolle et al., 2000). Based on general stress theory, stress may be caused by negative emotions resulting from cybervictimization experiences; however, negative emotions were not considered in this study. According to the stress-and-coping model (Lazarus and Folkman, 1984), an individual determines whether cybervictimization is a stressful event by going through two processes; first, evaluating whether the stressor (cybervictimization) is a threat; second, if it is a threat, evaluating whether there are sufficient skills or resources to deal with the stressor (cybervictimization). For some individuals, cybervictimization may be a threat, but they have sufficient skills or resources to deal with it; for others, cybervictimization may not be a threat. Hence, the mediating effect of stress would not be observed. Additionally, other studies have concluded that cyberbullying is a series of stressful events (Lianos and Mcgrath, 2018) and have emphasized the continuity of stressful experiences. However, this study only investigated cybervictimization without considering the severity or duration of the stressful event. Consequently, the mediating role of stress in the relationship between cybervictimization and cyberbullying would not be identified.

The second hypothesis, which proposed that rumination plays a mediating role in the relationship between cybervictimization and cyberbullying, was supported. This result was consistent with Malamut and Salmivalli (2021) findings on traditional bullying. Numerous studies have confirmed that cybervictimization positively predicts adolescents’ rumination (Feinstein et al., 2014; Zhong et al., 2015); this study further confirms that cybervictimization significantly predicts rumination among adolescents in China. Several studies have shown that rumination significantly predicts aggressive behavior in individuals (Zhu, 2014; Guerra and White, 2017). This study confirmed that cyberbullying, as a specific type of aggression, was positively predicted by rumination. There was an association among cyber-victimization, rumination, and cyberbullying, and experiences of cyber-victimization induced rumination, leading to retaliation in the form of cyberbullying. The model of victim schema (Rosen et al., 2009) explains that victims have difficulty regulating emotions, leading them to react aggressively to perceived threats. Rumination is maladaptive and can play an important role in the process of cybervictimization, which predicts externalizing problems such as cyberbullying.

The third hypothesis proposes the influence of stress and rumination in the relationship between cybervictimization and cyberbullying. The results demonstrated that stress and rumination played a chain-mediating role in the theoretical model. The results further validate the general aggression model. According to the stress response model, negative, stressful events encountered by individuals are the most direct cause of ruminative thinking (Robinson and Alloy, 2003). Life stressors can elevate adolescents’ rumination levels (McLaughlin et al., 2009), and rumination mediates the association between life stress and externalizing problems, such as aggressive behavior (LeMoult et al., 2019). The results of the current study can be better understood with response style theory (Nolen-Hoeksema, 1987), which states that individuals who adopt rumination repeatedly think about stressful events and pay constant attention to them, causing them to experience more intense stress, leading to aggressive behavior. Therefore, participants who were cyberbullied and repeatedly thought about the reasons for this bullying were more likely to treat others in the same way, that is, by committing cyberbullying.

## 5. Conclusion

This study investigated the relationship between cybervictimization and cyberbullying among college students, including the mediating roles of stress and rumination. The results show that cybervictimization not only directly affects cyberbullying but also has indirect effects through rumination and the chain mediating effect of stress and rumination. The Internet’s proliferation has led to the attack on Internet users and has made it inevitable for users to be cyberbullied or engage in cyberbullying.

The findings indicate that college students, first, experience cybervictimization before engaging in cyberbullying. The stress associated with their cyberbullying experience affects their way of thinking and makes them retaliate violently by engaging in cyberbullying to relieve themselves from the stress resulting from their bullying experiences. This behavior may be due to the fact that parents pay much attention to grades obtained by their adult children but disregard their psychological well-being, which makes the college students to bear the pain of cyberbullying alone without disclosing it to their parents or to their teachers or friends for a solution. This phenomenon is applicable to all cultures; therefore, it is necessary that teachers, parents, or friends, should provide proper guidance to college students who experience cyberbullying to relieve them from stress and change their negative attitude towards cyberbullying, as well as prevent them from self-blame and from becoming cyberbullies in retaliation to their bully experiences. Similarly, Internet platforms should be designed in such a way that they can actively block the occurrence of cyberbullying and make the Internet better for all. We suggest that future research should investigate the influencing factors and macro systems between cybervictimization and cyberbullying, and investigate factors that inhibit cyberbullying from cultural norms, social help, and family protection, while establishing a tripartite cyberbullying intervention system and measures for schools, society, and families.

### 5.1. Limitations

As with all research, this study has some limitations. First, all data in this study were reported by participants, and thus its validity may be influenced by social desirability. Future research should attempt to use a variety of perspectives and collect data from peers, online social platforms, and strictly controlled experiments to improve data validity. Second, this study investigated only a certain time period; therefore, the temporal effect on the variables cannot be determined. Future studies should include a longitudinal design to examine the relationship between cybervictimization, stress, rumination, and cyberbullying over longer periods. Finally, this study only examined individual-level variables. The interaction of multi-layer environmental systems shapes individuals’ online experiences (Bronfenbrenner, 1979; Bronfenbrenner and Morris, 2007; O’Neill and Dinh, 2015), which may influence the hazard and protective factors associated with cybervictimization (Livingstone and Helsper, 2013; Tsitsika et al., 2014; O’Neill and Dinh, 2015). Future research should consider multiple levels of influencing factors.

## Data availability statement

The original contributions presented in the study are included in the article/supplementary material, further inquiries can be directed to the corresponding author.

## Ethics statement

The studies involving human participants were reviewed and approved by Scientific Review Committee of the School of Public Policy and Administration, Nanchang University. The patients/participants provided their written informed consent to participate in this study.

## Author contributions

QL contributed to the experimental design, analyzed the data, and drafted the manuscript. NW helped revise the manuscript. LH provided final approval of the manuscript. All authors contributed to the article and approved the submitted version.

## Funding

This study was supported by The National Social Science Foundation of China(Grant no. 21CKS037), The Jiangxi Provincial Social Science Thirteenth Five-Year Plan Planning Youth Project (Grant no. 17JY32) and The Humanities and Social Sciences Research Planning Youth Project for Universities and Colleges in Jiangxi Province (Grant no. XL18106).

## Conflict of interest

The authors declare that the research was conducted in the absence of any commercial or financial relationships that could be construed as a potential conflict of interest.

## Publisher’s note

All claims expressed in this article are solely those of the authors and do not necessarily represent those of their affiliated organizations, or those of the publisher, the editors and the reviewers. Any product that may be evaluated in this article, or claim that may be made by its manufacturer, is not guaranteed or endorsed by the publisher.
